# Possibility to Alter Dynamics of Luminescence from Surface of Polymer Membrane with Ultrasonic Waves

**DOI:** 10.3390/polym14132542

**Published:** 2022-06-22

**Authors:** Nikolai F. Bunkin, Maxim E. Astashev, Polina N. Bolotskova, Valeriy A. Kozlov, Artem O. Kravchenko, Egor I. Nagaev, Maria A. Okuneva

**Affiliations:** 1 Department of Fundamental Sciences, Bauman Moscow State Technical University, 2-nd Baumanskaya Street 5, 105005 Moscow, Russia; bolotskova@inbox.ru (P.N.B.); v.kozlov@hotmail.com (V.A.K.); ikrav514@gmail.com (A.O.K.); neonlight0097@gmail.com (M.A.O.); 2 Prokhorov General Physics Institute of the Russian Academy of Sciences, Vavilova Street 38, 119991 Moscow, Russia; astashev@yandex.ru (M.E.A.); nagaev_e@kapella.gpi.ru (E.I.N.)

**Keywords:** luminescence spectroscopy, polymer membrane, Nafion, ultrasonic irradiation, dynamic light scattering, deuterium-depleted water, absorption of ultrasound, acoustic flows, microrheology, bulk viscosity, shear viscosity

## Abstract

The temporal dynamics of luminescence from the surface of Nafion polymer membranes have been studied. In fact, the polymer membrane was soaked in liquids with different contents of deuterium. The test liquids were ordinary (natural) water (deuterium content equal to 157 ppm) and deuterium-depleted water (deuterium content is equal to 3 ppm). Simultaneously with the excitation of luminescence, the Nafion plate was irradiated with ultrasonic pulses, having a duration of 1 μs. The ultrasonic waves were generated with different repetition rates and amplitudes, and irradiated the surface of Nafion in the geometry of grazing or normal incidence. Luminescence regimes were studied when the membrane was irradiated with one ultrasonic wave (one piezoelectric transducer) or two counter-propagating waves (two piezoelectric transducers). It turned out that ultrasonic waves, which fall normal to the membrane interface, do not affect the dynamics of luminescence. At the same time, in the case of ultrasonic irradiation in the grazing incidence geometry, sharp jumps in the luminescence intensity occur, and the behavior of these jumps substantially depends on the mode of irradiation: one or two piezoelectric transducers. This allows for control of the dynamics of luminescence from the polymer surface. In accordance with this model, the possibility of altering the luminescence dynamics is due to the effect of unwinding the polymer fibers from the surface toward the liquid bulk upon soaking. It is important that such unwinding does not occur in deuterium-depleted water, which was confirmed in a direct experiment with dynamic light scattering from polydisperse aqueous suspensions of Nafion nanometer-sized particles; these suspensions were prepared in ordinary water and deuterium-depleted water. Thus, ultrasonic irradiation affects the dynamics of luminescence only when Nafion is swollen in ordinary water; in the case of deuterium-depleted water this effect is missed.

## 1. Introduction

The specific features of water interaction with hydrophobic and hydrophilic polymer membranes is currently a hot topic in various fields of physical chemistry. Just as challenging is the physical nature of the nanostructures that are formed inside the polymer matrix. For background on Nafion™ and its properties, see the review in [1]. Nafion is manufactured via the copolymerization of a perfluorinated vinyl ether comonomer with tetrafluoroethylene (see [1]). Its chemical formula is given below:
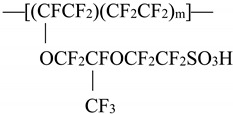


The legend to this formula is as follows: tetrafluoroethylene–perfluoro–3,6–dioxa–4–methyl–7–octenesulfonic acid copolymer.

Teflon is a highly hydrophobic matrix, while the sulfonic groups are very hydrophilic moieties. Therefore, when Nafion is soaked in water, the possibility of direct and reverse micellization appears (see [1]). Such a possibility is of great interest from a fundamental point of view, and also opens up a number of interesting applications in various industrial fields. This is why the number of publications devoted to the study of Nafion is constantly growing (see, e.g., [2,3,4,5,6,7,8,9], related to the articles dedicated to the study of various properties of Nafion and issued in 2022).

The Nafion matrix is biocompatible and flexible, and has excellent mechanical and chemical stability. When studying the specific features of Nafion, which are manifested during soaking in water, it is necessary to take into account the dissociation of terminal sulfonic groups at the polymer–water interface. Chemical formula of this reaction is R−SO_3_H + Н_2_О ⇔ R−SO_3_^−^ + H_3_О^+^. In addition, it is necessary to take into account the formation of through channels with a diameter of 2–3 nm in the bulk of the polymer matrix (see [1] for more details). Negatively charged areas at the inner surface of these channels provide a possibility for cations to transit through the bulk of the membrane [10,11,12,13]. Furthermore, the nanometer-sized structure of these channels allows for the separation of Н^+^ and ОН^−^ ions from both sides of the membrane, which is used in low-temperature hydrogen power plants [14]. The physical mechanism of such separation has been comprehensively studied; see, for example, the review in [15]. Intuition suggests that due to the mass conservation law, the formation of nanometer-sized channels should be accompanied by the unwinding of polymer fibers into the liquid bulk.

As is known, the luminescence spectroscopy is an effective technique for the investigation of polymers, see, for example, [16,17,18,19,20]. Certain features of Nafion swelling in various liquids by using this technique were investigated in [21,22,23,24,25]. As shown in [22], the luminescence centers are located on the terminal sulfonic groups. It turned out that the spatial distribution of the luminescence centers in the water bulk depends on the deuterium content of water in which the Nafion is being soaked. In fact, in an experiment in [22], the Nafion plate was soaked in water with different deuterium contents ranging from 1 ppm (the so-called deuterium-depleted water, DDW) to 10^6^ ppm (heavy water), and the size *x* of the area in the water bulk, adjoining to the interface and containing the luminescence centers, was measured. It was established that when Nafion is soaked in ordinary water (the deuterium content in ordinary (natural) water is 157 ± 1 ppm [26]), the luminescence intensity *I*(*x*) does not fall to the zero level up to the distance of *x* = 300 μm, while in the case of DDW, the intensity *I*(*x*) reached the zero value at *x* ≈ 5 μm. This result, taking into account the spatial resolution of our measuring device, allowed us to assume that in the case of ordinary water, the polymer fibers unravel toward the liquid bulk, while this effect is absent in DDW.

Studies of isotopic effects, revealed upon the soaking of Nafion, were further developed in [27]. In this work, it was found, by using the technique of Fourier transform IR spectroscopy, that the dynamics of swelling of Nafion in water in a cell of limited size (this size was essentially less compared to the size of the area, occupied with the unwound fibers in the luminescence experiments) also depends on the isotopic content of water. It turned out that in the case where the swelling occurs in ordinary water, a gas cavity between the Nafion plate and the cell window is formed; the size of this cavity is slightly smaller than the size of the Nafion plate. At the same time, when swelling in DDW, no cavity is formed. In [27,28], it was found that the appearance of such a cavity in ordinary water was due to the unwinding of initially hydrophobic polymer fibers toward the bulk of water. Since the size of the cell is less than the average length of unwound fibers, these fibers abut against the windows of the cell, and a field of local shear stresses arose, which, in turn, resulted in the extrusion of water molecules from the gap between the unraveled polymer fibers (i.e., a gas cavity, free of water molecules, should be formed). In the framework of this model, it is clear that such a cavity cannot appear during swelling in DDW. However, the conclusion about such unraveling was made on the basis of indirect experimental data; this effect has not yet been confirmed in a direct experiment.

Concluding this section, it is worth mentioning one additional manifestation of the unwinding effect. Namely, in [23], the swelling of the polymer membrane in ordinary water/DDW was studied upon irradiation by a low-frequency electromagnetic wave. It was found that random oscillations of the luminescence intensity occurred upon soaking in ordinary water, while this effect is missed upon soaking in DDW. According to the model developed in [23], this effect can be explained as follows. Let us first imagine that there is a luminescence center, localized on the membrane surface, which will be termed as a donor (see monographs [29,30,31,32] for more details). Let us further assume that there exists another particle (acceptor) at a certain distance *r* from the donor; the absorption spectrum of the acceptor coincides with the absorption spectrum of donor. As above-mentioned, the luminescence centers are terminal sulfonic groups, which can be negatively charged due to dissociation, or these remain electrically neutral in the absence of dissociation. The issue of which sulfonic groups, charged or electrically neutral, are the centers of luminescence, was not investigated in [22]. It is clear, however, that charged and neutral sulfonic groups must have the same structure of quantum levels.

As is known [29,30,31,32], if the quantum levels for donors and acceptors are the same, the resonant energy transfer of the luminescent state between such particles becomes possible. It is very important that this transfer occurs at a certain distance *r* between the donor and acceptor. The theory of energy transfer is based on the concept of a fluorophore as an oscillating dipole, which can exchange energy with another dipole, having a similar resonance frequency [32]. In fact, an electron from the excited acceptor level can radiatively transit into the ground state of the acceptor, which is accompanied by a photon emission (luminescence), but a non-radiative transition is also possible. In the latter case, the luminescence appears to be quenched. It is clear that the spatial contact of the electrically neutral and charged sulfonic groups is accompanied by electron transfer, which, in turn, should result in the luminescence quenching (see, for example, monograph [33]). Thus, the transfer of resonant energy from a charged to electrically neutral sulfonic group (or vice versa) is evidently related to the non-radiative transition.

The efficiency *E* of resonant energy transfer from a donor to an acceptor is given by formula

(1) $$E=\frac{R_{0}^{6}}{R_{0}^{6}+r^{6}}$$

Here, *R*_0_ = 20–60 Angstroms is the so-called Förster parameter (see [34]). It is seen that the value of *E* depends on *r* as *r*^−6^ (i.e., it is a very steep function). If the donor and acceptor are rigidly fixed on the membrane surface, then the distance *r* between them is constant (i.e., the efficiency *E* does not change upon swelling, see [35]). In this case, the luminescence intensity *I*(*t*) can be approximated by the formula [22],

(2) $$I(t)=A_{0}+\kappa I_{pump}\sigma_{lum}n_{Naf}(t)$$

Here, *I_pump_* is the pump intensity; *n_Naf_* is the near-surface volume number density of luminescence centers (the sulfonic groups); σ*_lum_* is the luminescence cross-section; *A*_0_ ~ 20–270 arb. units, which is related to a stray light; *κ* is a dimension coefficient, which stands for the spectrometer sensitivity; and *V* is the luminescence volume.

In the case where *E* is constant, the luminescence cross section σ*_lum_* is also constant. Thus, the near-surface sulfonic group density in the bulk of membrane close to its surface decreases upon swelling (liquid molecules penetrate into the near-surface layer, which results in reducing the density of the luminescence centers), and the equation for the sulfonic group density:

(3) $$\frac{dn_{Naf}}{dt}=-\frac{n_{Naf}}{\tau },$$

where *τ* is the characteristic time of the penetrating liquid molecules inside the polymer matrix. The solution to Equation (3) is as follows:

(4) $$n_{Naf}\left(t\right)={\left(n_{Naf}\right)}_{0}\exp\left(-\frac{t}{\tau }\right)$$

Since *I*(*t*) ∝ *n_Naf_*(*t*), the luminescence intensity *I*(*t*) obeys the exponential law of decay.

At the same time, if the distance *r* between the donor and the acceptor changes during soaking, that is, the value of *E* is not constant, then the luminescence cross section σ*_lum_* should also change with time. In this case, the dependence *I*(*t*) will no longer be described by a decaying exponential function. The question arises of a possibility to control the distance *r* between the donor and acceptor upon swelling. If this is possible, then this should be manifested in experiments on the excitation of luminescence. Altering the effective distance *r* between the donor and acceptor seems to be possible if the donor (sulfonic group) and acceptor (modified sulfonic group) are located on the polymer fibers unwound in the liquid bulk (i.e., their mutual spatial location can be changed due to some external influences. If the acceptor is not luminescent-active (quantum transitions for an acceptor are non-radiative), then at the value *E*~1, the luminescence is terminated (quenching effect). At the same time, if *E* << 1 (i.e., there is no energy transfer from the donor to the acceptor), the luminescence is quite intense.

The basic motivation of this work was to confirm that the effect of Nafion fiber unwinding does exist in a direct experiment. For this, experiments on the dynamic light scattering from Nafion nanometer-sized particles were conducted. The Nafion particles, suspended in ordinary water or DDW, were studied in this experiment. It was also interesting to find out whether it is possible to alter the luminescence dynamics when Nafion is swollen in ordinary water and DDW. Since the luminescence intensity is controlled by the value of *E*, and in the case where the distance *r* between the donor and acceptor can be changed, for example, with the help of ultrasonic wave, then the possibility of altering the luminescence dynamics (and, possibly, the swelling itself) can be realized. In the work presented below, the excitation of luminescence from the surface of the Nafion during swelling in ordinary water and DDW together with irradiation with ultrasonic waves was studied.

## 2. Materials and Methods

The Nafion N117 plates (Sigma Aldrich, St. Louis, MO, USA) with a thickness of 175 μm and an area of 1 × 1 cm^2^ were investigated The test liquid was deionized water (deuterium content 157 ± 1 ppm) with a resistivity of 18 MΩ × cm at 25 °C, refined by a Milli-Q apparatus (Merck KGaA, Darmstadt, Germany). Another test liquid was deuterium depleted water (DDW, deuterium content ≤ 1 ppm), purchased from Sigma Aldrich, St. Louis, MO, USA.

**Figure 1:**
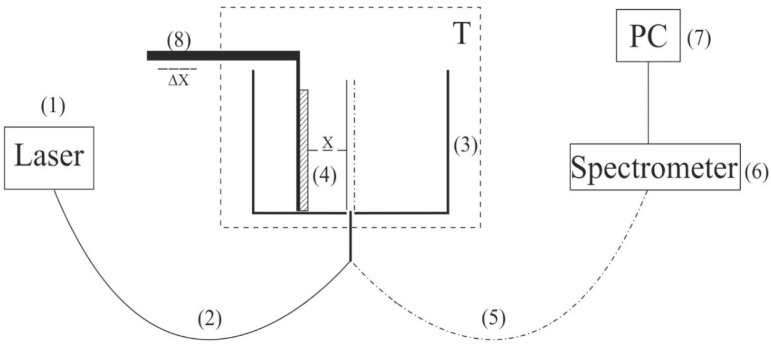
A schematic of the experimental setup: (1) continuous wave laser diode, (2, 5) multimode optical fibers, (3) cell with liquid sample, (4) Nafion plate, (6) spectrometer, (7) computer, (8) stage with horizontal micrometric feed for aligning Nafion plates with respect to the optical axis, and (T) is thermostat.

The experimental setup is exhibited in Figure 1. This setup includes a continuous wave diode laser (1) with a wavelength λ = 369 nm; spectrometer (6) with a spectral range of 240–1000 nm and resolution of 2 nm; multimode optical fiber (2) with a diameter of 50 μm, which transfers laser radiation (1) to cell (3) with a liquid sample; and similar optical fiber (5), through which a luminescence signal is fed to the spectrometer input. To prevent fairly intense laser radiation from arriving at the input of spectrometer (6), the fiber (5) was equipped with a refocuser and light filter, which cuts off radiation in the range λ < 350 nm (is omitted in Figure 1). Spectra were processed with a personal computer (7). Cylindrical cell (3) was made of Teflon and had a radius of 2 cm. Nafion plate (4) was mounted on stage (8) with a horizontal micrometric feed. This feed was used for exact alignment of Nafion plate with respect to optical axis, which coincided with the geometric axis of cylindrical cell. Thus, the Nafion plate was exposed to the laser beam in the grazing incidence geometry.

The laser diode radiation stimulated luminescence from the Nafion surface in the spectral range from 400 to 600 nm. As was demonstrated in [36], when a Nafion plate is immersed in water, surface bundles of polymer fibers tend to be oriented perpendicular to the surface (i.e., terminal sulfonic acid groups are located mainly at the polymer–water interface). This is why the luminescence was excited in the grazing incidence geometry of the pump. Since Nafion is transparent for visible light, the luminescence radiation, being reflected from the cylindrical cell wall, was concentrated along the optical axis and then arrived at the input of spectrometer (6).

Prior to the experiment, the luminescence spectrum of dry Nafion was measured; in this case, the cell was not filled with a liquid sample. The Nafion plate was oriented with the micrometer screw of stage (8) to make the luminescence signal in its spectral maximum (460 nm) as high as possible, which corresponds to the optimal orientation of the Nafion plate along the optical axis. Next, a test liquid was poured into the cell; the instant of the cell filling corresponded to the Nafion swelling onset. During swelling, the thickness of the Nafion plate increases (i.e., the “polymer–liquid” boundary shifts slightly across the pump beam). This is why the plate was rigidly fixed with special holders made of stainless steel (i.e., the thickness changing due to swelling was minimized).

In this experiment, it was possible to irradiate the Nafion surface with an ultrasonic wave. For this purpose, a G5-63 generator of electric pulses (Priborelektro, Moscow, Russia) and an oscilloscope AKIP 4115/3A (Novapribor, Moscow, Russia) were used. Furthermore, one or two piezoelectric transducers of a thickness *d* = 350 μm, made of piezo-electric ceramics Pb(Zr*_x_*Ti_1−*x*_)O_3_, were used; here *x* is the mole fraction, *x* ≈ 0.48. These transducers were mounted on a special holder in such a way that the ultrasonic wave could be directed either normally or in parallel to the Nafion surface (grazing incidence); these transducers are not shown in Figure 1. When attaching transducers, special attention was paid to the absence of elements that could absorb an ultrasonic wave.

**Figure 2:**
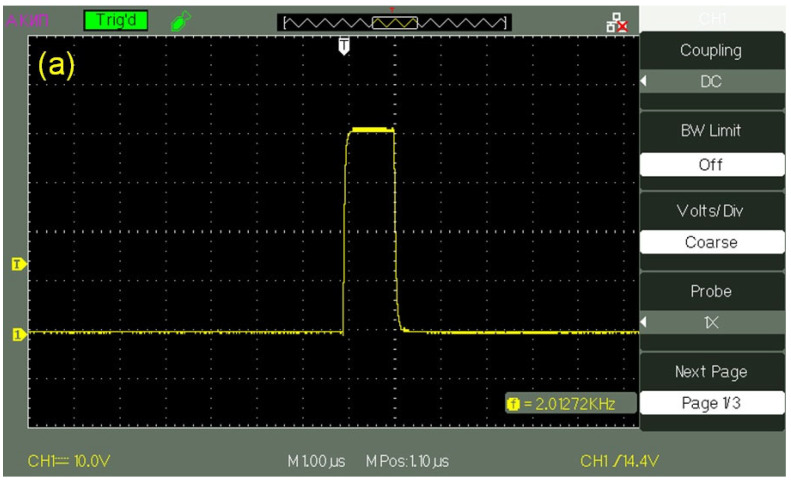

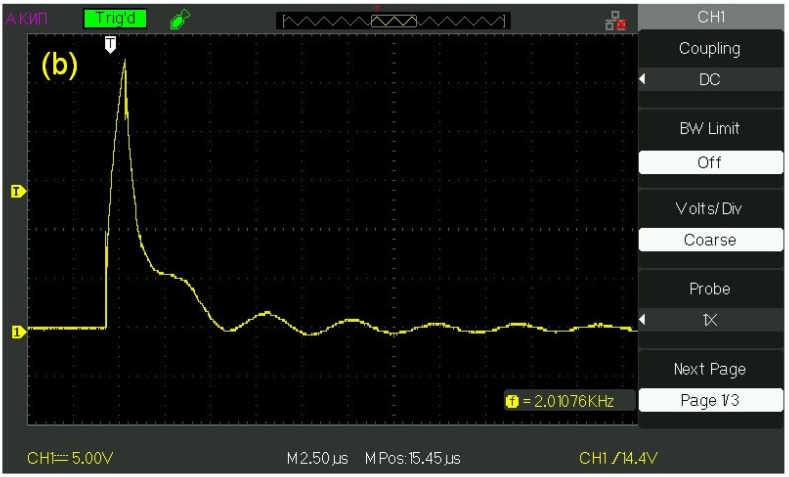
The oscillograms of electrical pulses that were fed to a piezoelectric transducer. Panel (**a**)—the transducer is not immersed in water. Panel (**b**)—the transducer is immersed in water.

The transducers were supplied with a pulsed voltage with an amplitude *U*_0_ = 60 V and a pulse duration in the range from 1 to 500 μs; Figure 2a exhibits an oscillogram of a pulse with a duration of 1 μs, and Figure 2b shows the oscillogram of this pulse, applied to the transducer, when the transducer is immersed in water. It can be seen that the initial rectangular shape of the pulse changed, and the pulse amplitude became equal to *U* = 25–27 V. These changes are obviously associated with the effective conductivity of the water layer. This layer can be considered as a low-resistivity element, which connects the electrodes of a piezo-ceramic transducer. The pulse repetition frequency varied from tens of Hz to hundreds of kHz. Assuming that the velocity of longitudinal sound in Pb(Zr_0.48_Ti_1−0.48_)O_3_ piezo-ceramics is *C_sound_* = 4.32 × 10^3^ m/s (see monograph [37]), and supposing *d* = λ/2, where λ is the wavelength of longitudinal sound (this follows from the boundary conditions for sound emission in the case of free boundaries of emitter), the ultrasound frequency ν = 6.1 MHz.

To make sure that when Nafion is soaked in ordinary water, the polymer fibers unravel toward the water bulk, while this effect is absent in DDW, the sizes of the Nafion nanometer particles in aqueous suspensions based on ordinary water and DDW have been measured. To carry out such an experiment, a Nafion sheet (recall that the thickness of this sheet was 175 μm) was first cut to the particles of about 1 mm in size, and then these particles were ground in a ball mill MSK-SFM-12M Milling Machine (MTI Corporation, Richmond, CA, USA) to obtain a polydisperse powder. To find the sizes of the resultant Nafion particles in aqueous suspensions based on ordinary water and DDW, dynamic light scattering (DLS) experiments with a Zetasizer Ultra/Pro setup (Malvern, UK) was carried out. The DLS technique was comprehensively described in monographs [38,39]. The DLS device was equipped with a continuous wave He − Ne laser at a wavelength of λ = 633 nm (maximum intensity is 4 mW) and a temperature controller; the scattering angle was 173°.

## 3. Experimental Results

**Figure 3:**
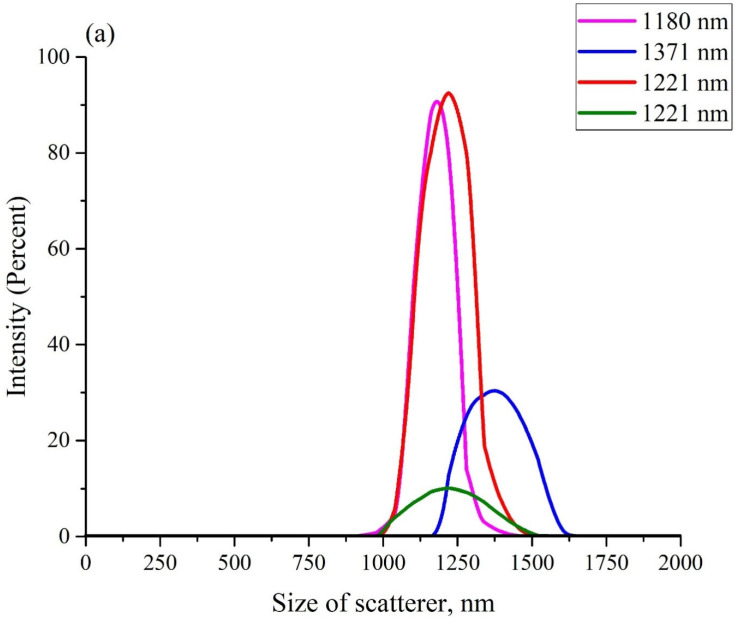

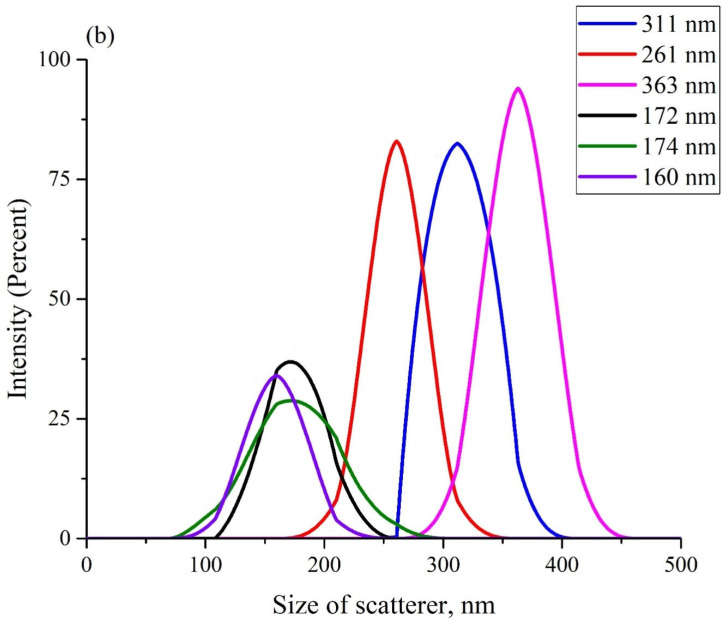
The distributions of scattering intensity over the sizes of the scatterers. Panel (**a**)—ordinary water. Panel (**b**)—DDW.

Figure 3 exhibits the scattering intensity distributions over the sizes of Nafion particles in aqueous suspensions in DDW (panel (a)) and in ordinary water (panel (b)). These suspensions should apparently contain particles with a diameter in the range from hundreds of nanometers to units (and, possibly, tens) of microns. As is known [40], it is impossible in DLS experiments to detect particles with sizes larger than 6–7 μm (i.e., particles with such sizes did not manifest in anyway in the plots in Figure 3). The scattering intensity distributions, obtained in each particular experiment, are highlighted with different colors. The insets in the graphs show the effective sizes of the scattering particles (maxima of distributions). The ordinate axis shows the percentage of scatterers with a given effective size, which was detected in each particular experiment.

Before each measurement, the liquid sample was vigorously shaken. The fact that the particle sizes, obtained in different experiments, differed from one another, could be associated with the polydispersity of the Nafion powder. It is important that in all experiments, the size of the Nafion particles in ordinary water (panel (a)) was several times larger compared to the particles in DDW (panel (b)). However, there is no reason to believe that the sizes of particles in the Nafion powder, which appeared in ordinary water, significantly exceeded the sizes of particles in DDW. Thus, the only mechanism for the increase in the particle size in ordinary water is due to the unraveling of the polymer fibers, that is, the effect of unwinding in ordinary water and the absence of this phenomenon in DDW was confirmed in a direct experiment.

The next series of experiments was dedicated to the study of ultrasonic irradiation of the Nafion polymer membrane, which was soaked in ordinary water and DDW. In these experiments, ultrasonic irradiation was directed either normally to the membrane surface, or along the surface (grazing incidence geometry). Furthermore, either one piezoelectric transducers or two (in the last case, two ultrasonic waves propagate toward each other) were used. Note that irradiation with ultrasound normal to the membrane surface did not result in any visible changes in the behavior of the luminescence intensity *I*(*t*). This is why the results of the experiments with such geometry are not presented.

**Figure 4:**
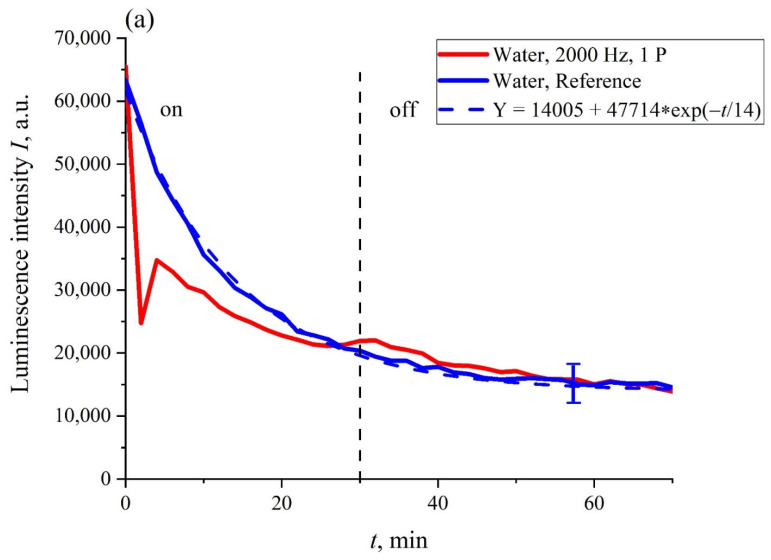

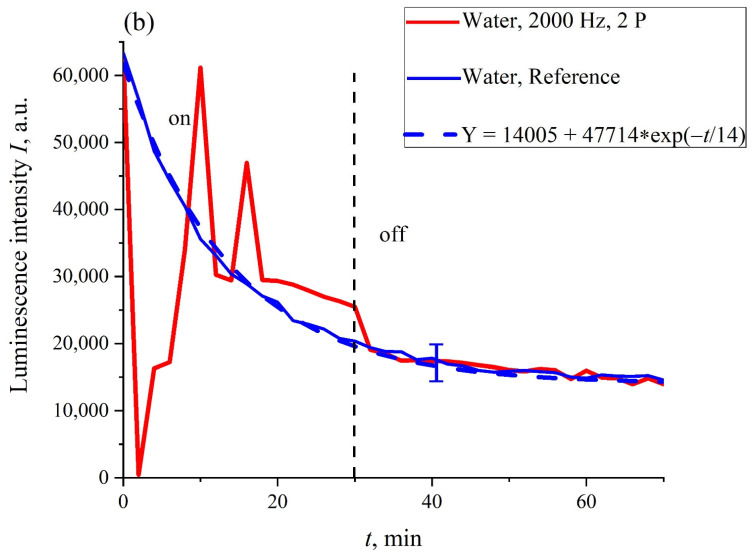
The dependencies of the luminescence intensity *I*(*t*) on the soaking time in ordinary water upon ultrasonic irradiation with one piezoelectric transducer (panel (**a**)), and two transducers (panel (**b**)). The vertical dotted line marks the moment when the ultrasound was turned off (30th minute).

In Figure 4a,b the most typical dependences of the intensity of luminescence *I*(*t*) vs. the soaking time *t*, are exhibited. The experimental curves are related to Nafion membrane swelling in ordinary water during irradiation with one piezoelectric transducer (panel (a)) and two transducers (panel (b)) for a pulse repetition rate of 2 kHz (oscillograms of such pulses are shown in Figure 2a,b in the grazing incidence geometry. The dependences for non-irradiated water (reference dependences) are also shown. It can be seen that the reference curves were well approximated by exponential functions (see the comments to Equation (4)). The vertical dotted line marks the moment when the ultrasound was turned off (30th minute). It was seen that in the case of one piezoelectric transducer, the moment when the ultrasound was turned off practically did not manifest itself in the dynamics of *I*(*t*), while in the case of two piezoelectric transducers, the dependence of *I*(*t*) approached the reference dependence after the ultrasound had been turned off. The reference dependencies were able to be reproducible quite well; the confidence intervals for these curves are given. Note that the dependencies *I*(*t*) obtained upon irradiation with ultrasound in ordinary water were not reproduced. This is why only fairly typical graphs are presented.

**Figure 5:**
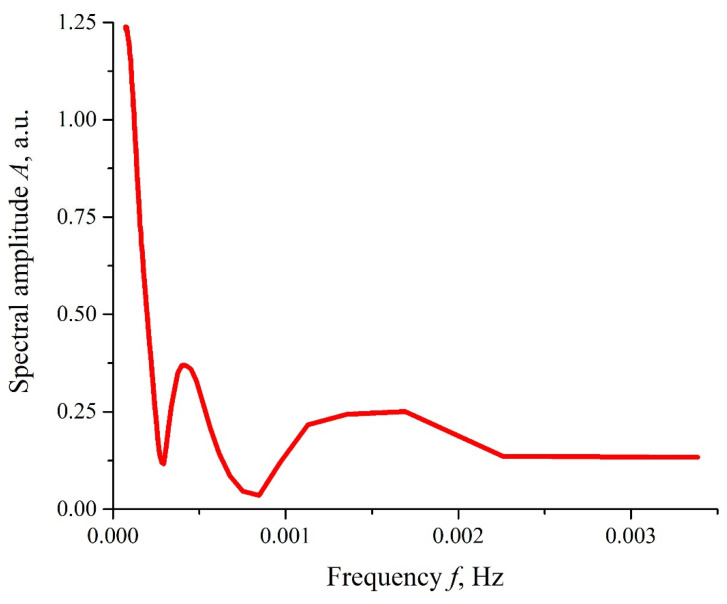
The frequency dependence of the spectral amplitude, *A*(*f*), for the *I*(*t*) plot shown in Figure 4b.

As follows from the graphs in Figure 4, the most significant differences between the dependencies *I*(*t*) and the reference curves were observed for the irradiation of the Nafion membrane with two piezo-ceramic transducers that emit ultrasonic waves toward each other (panel (b) in Figure 4). In Figure 5, the dependence *A*(*f*), which is the spectral amplitude at frequency *f*, is shown. This dependence was obtained via the Fourier transform of the function *I*(*t*) shown in Figure 4b, red curve. The Fourier transform was performed by using the Morlet wavelet (see [41]). In this particular case, the Morlet wavelet is given by the formula $\psi \left(t\right)=\exp\left(-t^{2}/2\right)\cos\left(5t\right).$ It can be seen that there exist two local spectral maxima in the frequency range 10^−4^−10^−3^ Hz; the commentary to this graph will be given in the next section. It was also seen that, as frequency *f* → 0, the dependence *A*(*f*) diverges, which is related to the uncontrolled contribution of low-frequency random processes. Since the physical nature of these low-frequency processes is unknown, any comments on this are omitted.

**Figure 6:**
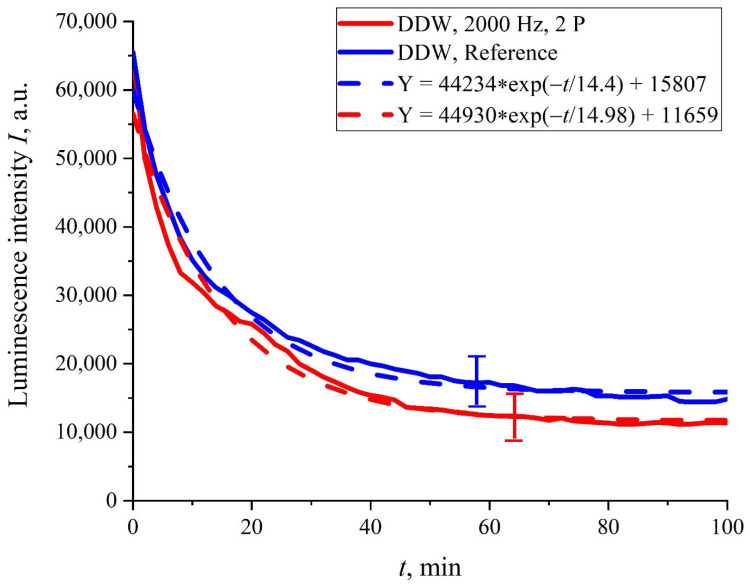
The intensity of the luminescence *I*(*t*) vs. soaking time *t* in DDW upon ultrasonic irradiation with two piezoelectric transducers.

It turned out that in the case where the Nafion membrane swelled in DDW, the dynamics of *I*(*t*) were not sensitive to ultrasonic irradiation, regardless of whether one or two piezoelectric transducers were applied. In Figure 6, the dependence *I*(*t*) for the case of two transducers is presented. It can be seen that in this case, there were not any features in the run of the *I*(*t*) curve, and this dependence was well-reproduced (confidence intervals and an approximating exponential function are indicated). In addition, the curve *I*(*t*) was close to the reference curve, which was confirmed by overlapping the confidence intervals and the proximity of the corresponding exponential functions (see the inset in Figure 6).

## 4. Discussion

As follows from the results reported in the previous section, the effect of ultrasonic irradiation of the polymer membrane on the dynamics of luminescence manifest itself when the membrane swelled in DDW. As follows from the results did not presented in the graphs in Figure 3a,b, the effect of unraveling of polymer fibers toward the bulk of the liquid took place in ordinary water, while in DDW, this effect was absent. Thus, it is straightforward to assume that the effects of ultrasonic irradiation in the grazing incidence geometry were precisely due to such unraveling. Before explaining the obtained results, it is necessary to completely describe the sound field. When an external voltage *U* is applied to the plates of a piezo-ceramic capacitor with a capacitance *C* and a volume *V*, longitudinal vibrations of the capacitor surface with a speed *v* due to the piezoelectric effect occur. The transfer of energy from electrical vibrations to ultrasound is described by the formula (see monographs [42,43,44]) below:

(5) $$K^{2}\frac{CU^{2}}{2V}=\frac{\rho v^{2}}{2}$$

The material constants included in Equation (5) for the piezo-ceramics [Pb(Zr,Ti)O_3_], were found in [45,46,47,48]. Here, *K* = 0.2 is the electromechanical coupling factor, *ρ* = 5.3 × 10^3^ kg/m^3^ is the density of piezo-ceramic, *ε* = 1500 is its dielectric constant. The transducer capacitance is $C=\frac{\varepsilon_{0}\varepsilon S}{d}$, where *d* = 350 μm is the thickness of the transducer; *V* = *Sd* is its volume; and *v* = *A*ω is the velocity of the transducer surface oscillations, where *A* is the amplitude of the ultrasonic wave.

Since ω = 2πν = 6.1 × 6.28 = 38 MHz (see the second Section), *ε*_0_ = 9 × 10^−12^ F/m, *U* = 27 V (see. Figure 2b), the amplitude is expressed as$A=\frac{KU}{\omega d}\sqrt{\frac{\varepsilon_{0}\varepsilon }{\rho }}=6$Å ≈ 1 nm (i.e., *v* ≈ 3.8 × 10^−2^ m/s). For a longitudinal ultrasonic wave to be capable of changing the dynamics of the luminescence intensity *I*(*t*), it is necessary that the condition *А* > *R*_0_ should be met, where *R*_0_ = 2–6 nm is the Förster parameter (see the Introduction Section, and also [34]). Indeed, only in this case, the luminescent energy can be resonantly transferred from a donor to an acceptor (which, according to the described above model, are located at the ends of unwound polymer fibers). However, in accordance with the estimates, the condition *А* > *R*_0_ is not satisfied. Thus, the peculiarities in the behavior of *I*(*t*), observed in the graphs of Figure 4, cannot be due to the vibrating of polymer fibers excited by ultrasonic wave.

As follows from Figure 5, the spectral amplitudes *A*(*f*) of the luminescence intensity *I*(*t*) belong to the frequency range 10^−4^–10^−3^ Hz, that is, *I*(*t*) is a slow function of time. In order to describe the slow changes in *I*(*t*), it is necessary to take into account the absorption of the longitudinal ultrasonic wave inside the near-surface layer containing the unwound polymer fibers. The absorption of an acoustic wave gives rise to the so-termed acoustic streaming, which is a steady flow in a fluid. A sound wave passing through an absorbing medium carries an impulse, which is gradually transferred to the particles of liquid, causing their movement in the direction of the original sound wave. In other words, the nature of acoustic flows is explained in the framework of the momentum conservation law: due to absorption, the mechanical impulse of an acoustic wave is transferred to a viscous medium, and hydrodynamic flows with a velocity *u* are generated in the liquid (see [49,50,51,52] for more details). It is important that this velocity depends on the intensity of sound and the viscosity of the medium.

In the case under study, acoustic (hydrodynamic) flows manifest themselves differently for one and two piezoelectric transducers. In the situation of two transducers, which emit ultrasonic waves toward each other along the surface of the Nafion plate (see Figure 4b), two acoustic flows move to the center of the Nafion plate with the speed *u*. These flows effectively shift the ends of the unwound polymer fibers toward the central part of the Nafion plate. It is this region that is probed by the laser pump beam (i.e., the luminescence excitation occurs predominantly from this area). As follows from the graph in Figure 4b, the intensity *I*(*t*) the zero level 2 min after the start of soaking under sonication. The reduction in the fell to luminescence intensity is attributed precisely to a growth in the density of unwound polymer fibers (i.e., to a decrease in the effective distance between them). In this case, the distance *r* between a donor and an acceptor of luminescence, which are localized on the unwound fibers, essentially decreases, and the efficiency *E* of resonant energy transfer can be close to unity (see Equation (1)). This should result in the luminescence quenching.

For the quenching of luminescence, two counter-propagating hydrodynamic flows should be generated. This was confirmed by the graph in Figure 4a for the case where only one piezoelectric transducer was applied. In this case, the luminescence intensity *I*(*t*) also fell, but this decrease did not reach zero level, and the intensity *I*(*t*) upon sonication quickly approached the reference curve. It can be seen that there were no steep jumps in the behavior of *I*(*t*). In addition, in the case of one piezoelectric transducer, the moment when the ultrasound was turned off did not manifest itself in any way in the dependence *I*(*t*), while in the case of two piezoelectric transducers, the *I*(*t*) curve reached the reference curve precisely after the ultrasound was turned off.

Within the framework of this model, the velocity *u* = *L*/2τ was numerically estimated. Here, *L* = 1 cm is the size of the Nafion plate along the sound wave direction, and τ = 2 min (see Figure 4b; at this moment of time, the luminescence intensity fell to the zero level), in other words, *u* = 4 × 10^−5^ m/s. As follows from [49], there exist several modes of the propagation of acoustic flows, which differ from one another with respect to the value of *kl*_0_, where *k =* 2π/λ is the wavenumber and*l*_0_ is the characteristic scale of the flow. Since in the case of interest the sound wave propagated near the Nafion–water boundary inside the layer of unwound polymer fibers, it is straightforward to assume that the length of this layer is about *l*_0_(i.e., *l*_0_ ≈ 300 μm, see [22]). Under the condition *Ma*(*kl*_0_)^2^ << 1, where *Ma* = *v*/*С_sound_* is the Mach number, the velocity ratio *u/v* (here *v* = *Аω* ≈ 3.8 × 10^−2^ m/s is the velocity of particles in the wave) is given by the formula (see [52] for more details):

(6) $$u/v=\frac{1}{3}\left(1+\frac{3}{4}\frac{\eta_{V}}{\zeta }\right)Ma{\left(kl_{0}\right)}^{2}$$

Here, *η_V_* and *ζ* are accordingly the coefficients of the bulk and shear viscosity inside the layer containing the unwound polymer fibers, respectively. Bulk viscosity can be found from the formula

*η_V_* = *η*’ *+* (2/3)*η*, (7)

where *η*’ is the second viscosity and *η* is the dynamic viscosity (see [45] for more details). Note that for water, the coefficient of the second viscosity at room temperature is *η*’ = 3.1 × 10^−3^ Pa × s ≈ 3*η* (see [53], i.e., for water *η_V_* ≈ 3.7*η*).

Assuming that *С_sound_* = 1.5 × 10^3^ m/s is the speed of sound in water, the Mach number is *Ma* ≈ 2.5 × 10^−5^(i.e., the product *Ma*(*kl*_0_)^2^ ≈ 1.8 × 10^−4^), and the ratio *u/v* can be estimated from Equation (6). The numerical estimates were *u/v*~10^−3^, and *η_V_*/*ζ* ≈ 30. Unfortunately, it makes no sense to carry out further estimates, since the spatial distributions of the bulk and shear viscosities *η_V_* and *ζ* along the unwound polymer fibers are unknown. Note, however, that the presence of shear viscosity inside the layer containing the unwound polymer fibers allows us to discuss the micro-rheological properties of this layer (see monographs [54,55]). In a recent work in [56], some attempts to probe such micro-rheological properties of the Nafion membrane have been reported. The study in [57] described some of the techniques for measuring the shear viscosity coefficient *ζ* in the rheometry experiments. Apparently, the use of rheometric techniques together with irradiation with ultrasonic waves will make it possible to measure the coefficients *η_V_* and *ζ* separately.

Let us turn once again to the graphs in Figure 4b and Figure 5. After the first two minutes of Nafion swelling, the luminescence intensity *I*(*t*) approached the zero level, which, according to the model suggested, corresponded to the overlapping of two oppositely directed hydrodynamic flows in the region close to the center of the Nafion plate, which results in an increase in the density of the unwound fibers in this area. In this case, the efficiency *E* of thee resonant transfer of the luminescent energy from the donor to acceptor is about unity, and the luminescence intensity *I*(*t*) should decrease. Apparently, after the time τ = 2 min, the total velocity of hydrodynamic flows was *u* ≈ 0. In this case, the unwound fibers should return to their original state (i.e., the luminescence intensity should instantly increase, which indeed occurs, see Figure 4b). At the same time, we cannot rule out fluctuation jumps in the velocity *u*, possibly due to local turbulent vortexes in the counter-propagating flows. Thus, the jumps in the intensity *I*(*t*) at *t* > τ correspond to the fluctuations of the velocity *u* near the zero level. The dependence *A*(*f*) of the spectral amplitudes vs. the frequency, shown in Figure 5, stands for a low-frequency spectrum of such fluctuations at times *t* > τ.

Finally, within the framework of this approach, it is possible to explain on a qualitative level the absence of jumps in *I*(*t*) at the normal (with respect to the Nafion surface) incidence of ultrasonic waves. In this case, the unwound polymer fibers are directed along the hydrodynamic flow, so it is highly possible that the distance between the donors and acceptors, localized at the ends of the unwound fibers, also changes. Remind that the donors and acceptors are presumably located at the distance of *l*_0_ ≈ 300 μm from the Nafion surface, illuminated by the pump radiation (the optical axis is directed along the Nafion surface). The changes in the spatial distribution of unwound fibers could of course result in the jumps of *I*(*t*), but these jumps could hardly be detected in the luminescence experiments. Indeed, the core diameter of the optic fiber for the pump radiation was 50 µm (see Section 2), in other words, the pump beam diameter on the Nafion surface is also about 50 μm << *l*_0_. It is clear, however, that this issue requires a more comprehensive analysis both in theory and in the experimental studies.

Concluding this section, we can argue that by applying ultrasonic irradiation to a polymer membrane swelling in ordinary water, it is possible to control (alter) the dynamics of the luminescence intensity *I*(*t*). It is straightforward to assume that when the density of unwound polymer fibers can be driven by counter-propagating acoustic flows, moving from the edges of the membrane to its center (i.e., the density of these fibers also increases from the edges to the center), it is possible to control the process of the swelling of the polymer membrane.

## 5. Conclusions

Summing up, it was established in a direct experiment based on the DLS technique that when Nafion swells in ordinary water, the polymer fibers unwind toward the bulk of the liquid, while this effect does not occur in DDW. Based on the experimental fact that the centers (donors) of the luminescence are sulfonic groups, localized at the unwound polymer fibers, and also under the assumption that luminescence acceptors (modified sulfonic groups) are also located at these fibers, the hypothesis about the possibility of controlling the resonant energy transfer from the donor to acceptor was put forward. It turned out that the distance between the donors and acceptors can be altered with the help of emitting ultrasonic waves toward each other along the polymer membrane. These waves, being absorbed in a layer containing unwound fibers, transfer their momentum to the liquid particles, that is, two acoustic flows directed toward each other are generated. By measuring the speed of these flows, it is possible to estimate the ratios of the coefficients of the bulk and shear viscosity within the layer containing the unwound fibers.

## Data Availability

The data presented in this study are available on request from the corresponding author.
